# Rare Variants of Obesity-Associated Genes in Young Adults with Abdominal Obesity

**DOI:** 10.3390/jpm13101500

**Published:** 2023-10-16

**Authors:** Ahmad Bairqdar, Elena Shakhtshneider, Dinara Ivanoshchuk, Svetlana Mikhailova, Elena Kashtanova, Viktoriya Shramko, Yana Polonskaya, Yuliya Ragino

**Affiliations:** 1 Federal Research Center, Institute of Cytology and Genetics, Siberian Branch of Russian Academy of Sciences, Prospekt Lavrentyeva 10, 630090 Novosibirsk, Russia; ahmad15.berkdar@gmail.com (A.B.);; 2 Department of Genetics, Novosibirsk State University, Pirogova Str., 1, 630090 Novosibirsk, Russia; 3 Institute of Internal and Preventive Medicine, Branch of Institute of Cytology and Genetics, Siberian Branch of Russian Academy of Sciences, Bogatkova Str. 175/1, 630004 Novosibirsk, Russia

**Keywords:** abdominal obesity, obesity-associated genes, *ADIPOQ*, *FTO*, *GLP1R*, *GHRL*, *INS*, young adults, targeted sequencing technologies, rare variants

## Abstract

The increase in the prevalence of overweight, obesity and associated diseases is a serious problem. The aim of the study was to identify rare variants in obesity-associated genes in young adults with abdominal obesity in our population and to analyze information about these variants in other populations. Targeted high-throughput sequencing of obesity-associated genes was performed (203 young adults with an abdominal obesity phenotype). In our study, all of the 203 young adults with abdominal obesity had some rare variant in the genes associated with obesity. The widest range of rare and common variants was presented in *ADIPOQ*, *FTO*, *GLP1R*, *GHRL*, and *INS* genes. The use of targeted sequencing and clinical criteria makes it possible to identify carriers of rare clinically significant variants in a wide range of obesity-associated genes and to investigate their influence on phenotypic manifestations of abdominal obesity.

## 1. Introduction

The increase in the prevalence of overweight, obesity, and associated diseases is a serious problem for many countries [1]. In addition to the influence of environmental factors, the development of obesity is also facilitated by the presence of a genetic predisposition to gaining excess weight [2,3,4]. The monogenic type and syndromic types of obesity is determined with a frequency of 1:20,000–30,000 of newborns. The polygenic type of obesity occurs at different frequencies at different ages and in different populations [2]. Genetic factors may be involved in the formation of different types of obesity: syndromic, monogenic, and polygenic [2]. The monogenic and syndromic types of obesity usually develop in childhood and adolescence, the polygenic type at an older age.

The development of a monogenic type of obesity is caused by the presence of a pathogenic variant in the DNA sequence in one of the genes of the leptin-melanocortin system (*LEP*, *LEPR*, *POMC*, *PCSK1*, and *MC4R*). This type of obesity is extremely rare, characterized by onset in childhood and extreme values of obesity [5,6,7]. The leptin-melanocortin system is activated by leptin, which is secreted by adipocytes. The effect on the leptin receptor leads to the activation of pro-opiomelanocortin. Under the influence of the prohormone convertase 1 enzyme, adrenocorticotropic hormone and α-melanocyte-stimulating hormone are formed from proopiomelanocortin, which in turn activates the *MC4R* receptor, which leads to satiety signaling [5]. The leptin-melanocortin system is key in the regulation of eating behavior and energy metabolism [8]. Physical activity, socioeconomic status, and diet type may influence the severity of obesity in patients and the success of its treatment [7,9].

Patients with a number of genetic syndromes (Prader–Willi, fragile X, Bardet–Biedl, etc.) may develop a syndromic type of obesity accompanied by a complex of congenital developmental anomalies [7]. The clinical features of the syndromic type of obesity depend on the causative syndrome.

The polygenic type of obesity is widespread and develops as a result of the influence of many genes and their interaction both with each other and with environmental factors [10,11]. Excessive accumulation of visceral fat in the polygenic type of obesity leads to dysfunction of adipose tissue as an endocrine organ. With abdominal obesity, hypertrophy and hyperplasia of adipocytes develop, signs of an inflammatory process appear in the tissue, fibrosis develops, and the nature of adipokines secretion changes [12,13,14,15,16].

The prevalence of polygenic obesity increases with age [17,18], while young adults aged 25–44 years are studied less often. At a young age, abdominal obesity may be associated with clinically significant gene variants that cause polygenic obesity. The aim of the study was to identify rare variants in obesity-associated genes in young adults with abdominal obesity in our population and to analyze information about these variants in other populations.

## 2. Materials and Methods

### 2.1. Study Population

The screening of the population of residents aged 25–44 years of Novosibirsk (West-ern Siberia, Russia) was carried out in 2013–2016. The study involved materials from the “Collection of human biomaterials at the Institute of Internal and Preventive Medicine—a branch of ICG SB RAS” (No. 0324-2017-0048). The profile of the group of residents in the surveyed districts was typical for the city of Novosibirsk in terms of ethnicity, age, and employment status. From the Novosibirsk residents, a representative sample was chosen of 1512 subjects of 25–44 years old (males/females ratio 44/56, white ethnicity > 90%) using a random-number table. The study protocol was approved by the local Ethics Committee of the Institute of Internal and Preventive Medicine—a branch of the Institute of Cytology and Genetics (ICG), the Siberian Branch of the Russian Academy of Sciences (SB RAS), Novosibirsk, Russia, No. 6/2013 of 25 June 2013. From each patient, we obtained informed consent to be examined for the collection and analysis of biological samples.

### 2.2. Clinical Examination

The program of clinical examination included the registration of sociodemographic data; a standard questionnaire on smoking and alcohol use; a history of chronic diseases; the use of medications; the Rose cardiological questionnaire; anthropometric data (height, body weight, and waist circumference); three-time measurement of blood pressure; spirometry; electrocardiography; detection of “definite coronary heart disease” in accordance with validated epidemiological criteria (MI as determined by electrocardiography, pain-free coronary heart disease according to electrocardiography, or stable effort angina of functional classes II–IV according to the Rose questionnaire) and clinical-functional criteria (according to electrocardiograms interpreted via the Minnesota code); and biochemical assays of blood serum (total cholesterol, HDL-C, triglycerides, and fasting glucose).

### 2.3. Criteria for Inclusion in Genetic Study

In our study, 203 young people with abdominal obesity were randomly selected from the population for genetic study. Pregnant women and women on maternity leave were excluded. In Whites with a BMI ≥ 25.0 kg/m^2^ and in Asians with a BMI ≥ 23.0 kg/m^2^, waist circumference (WC) measurement is recommended as a simple and informative method for diagnosing abdominal obesity. Values of WC ≥ 80 cm in women and WC ≥ 94 cm in men correspond to abdominal obesity [19,20].

### 2.4. Blood Chemistry

Blood sampling from the cubital vein was performed in the morning on an empty stomach and at 12 h after a meal. Blood lipid profiling (total cholesterol, triglycerides, HDL-C, and LDL-C) was conducted via enzymatic methods using standard reagents (Biocon Fluitest; Lichtenfels, Germany) on a Labsystem FP-901 biochemical analyzer (Helsinki, Finland). The atherogenic coefficient was calculated using the formula: IA = (TC − HDL-C)/HDL-C. The serum glucose to plasma glucose conversion formula is as follows: plasma glucose (mmol/L) = −0.137 + 1.047 × serum glucose (mmol/L). The level of leptin and adiponectin was determined by multiplex analysis using the Human Adipokine Magnetic Bead Panel 1 kit (EMD Millipore Corporation, Darmstadt, Germany) on a Luminex 20 MAGPIX flow cytometer (Luminex Corporation, Austin, TX, USA).

### 2.5. DNA Extraction and Target Panel Design

Phenol-chloroform extraction was carried out to isolate DNA from the blood samples [21]. The quality of the extracted DNA was assessed using an Agilent 2100 Bioanalyzer capillary electrophoresis system (Agilent Technologies Inc., Santa Clara, CA, USA).

Gene selection for target sequencing was based on a review of the literature related to clinical features such as obesity and metabolic syndrome (central obesity, hyperglycemia, hypertriglyceridemia, Type 2 diabetes) and significant genetic variants associated with them [22] and included the genes (*ADIPOQ*, *ADRB3*, *APLN*, *APLNR*, *FTO*, *GCG*, *GLP1R*, *GHRL*, *GIP*, *INS*, *LEP*, *NAMPT*, *PPY*, *PYY*, *RETN*, and *SCT*). The target panel was designed using an available online tool (AmpliSeq, Illumina, San Diego, CA, USA) and included coding parts and adjacent splicing sites. The NGS library was prepared by hybridization capture with a (HiSeq SBS kit v4) reagent kit (Illumina, San Diego, CA, USA) following the manufacturers’ instructions. Target capture libraries were sequenced on the HiSeq 2000 platform (Illumina, San Diego, CA, USA), with 97% total coverage. The laboratory personnel performing the genotyping assays were blinded to the physical and clinical-examination data.

### 2.6. Bioinformatics Analysis

All sequenced reads were aligned to the human reference genome National Center for Biotechnology Information build 37 (GRCh37/hg19) using the Burrows-Wheeler Alignment tool (Ver. 0.7.17) [23]. PCR duplicates were removed using MarkDuplicates of PicardTools GATK (v.3.3) [24], and coverage was ×100. The ANNOVAR (ANNOtate VARiation, 24 October 2019) tool was used to call the genomic variants [25]. We estimated the pathogenicity of each novel variant according to the recommendations of the American College of Medical Genetics and Genomics (ACMG) and the Association for Molecular Pathology [26].

### 2.7. Statistical Analyses

The analyses of the data were carried out using the statistical software package IBM SPSS Statistics 22.0 for Windows (New York, NY, USA). The normal distribution of quantitative characteristics was determined by the Kolmogorov–Smirnov test. The distribution tested was normal. The significance level was set below 0.05.

## 3. Results

According to the results of a previous study, the prevalence of abdominal obesity in the main sample was 42.4% (in men—42.7%, in women—42.1%) [27]. In our study, individuals with abdominal obesity (n = 203) showed higher levels of BMI, total cholesterol, LDL-C, TG, and leptin (*p* < 0.05) (Table 1), which correlates with Ragino Yu.I. et al. for the main sample.

The search for rare variants was carried out in 16 genes associated with obesity. Rare variants have been identified in the *ADIPOQ*, *ADRB3*, *APLN*, *APLNR*, *FTO*, *GCG*, *GLP1R*, *GHRL*, *GIP*, *INS*, *LEP*, *NAMPT*, *PPY*, *PYY*, *RETN*, and *SCT* genes in young adults with abdominal obesity.

### 3.1. Variants in ADIPOQ

The common and rare variants of the *ADIPOQ* gene identified in this study are presented in Table 2.

The heterozygous intron variant rs17366653 in the *ADIPOQ* gene was identified in five young adults in our study. Previously, this variant was found to correlate with alternative splicing, in which isoforms with deleted Exons 2 and/or 3 were seven times more common in heterozygotes of rs17366653 than in samples of homozygotes of the reference allele [31]. A recent GWAS study identified a number of variants, including rs17366653, that correlate with decreased levels of adiponectin [32].

Another rare heterozygous variant rs199668131 in the intron of the *ADIPOQ* gene was identified by us in one patient (female; age 42 years; BMI 31.6; adiponectin level 4.14 μg/mL; high cholesterol and LDL levels). The missense variant rs143606172 was identified in one heterozygous carrier (female, age 26, BMI 26.26, high cholesterol, LDL, and TG). The missense variant rs62625753 was identified in three heterozygous carriers, all carriers had high levels of BMI, LDL and blood glucose. The missense variant in the heterozygous form rs17366743 was identified in one carrier (male, age 38 years, BMI 27) with high levels of cholesterol, LDL, and glucose. According to the literature, the frequency of this variant was higher in patients with DM2 [40]. The 3′UTR variant of rs4068 was identified by us in two heterozygous patients with overweight and high levels of cholesterol, LDL, and blood glucose.

Several common variants of the *ADIPOQ* gene are reported in the literature to be associated with obesity, T2DM, and metabolic syndrome, but these results are ethnospecific [39,41,42,43,44,45]. Three common variants, rs2241766, rs1501299, and rs17366743, were identified in the *ADIPOQ* gene in our study. According to the literature, homozygous carriers of the rs2241766 variant showed a higher risk of developing Type 2 diabetes and insulin resistance in the Japanese population; in addition, the GG genotype has been associated with T2DM in Chinese, Finnish, and Iraqi populations [33,46]. In the Mexican population, homozygotes for this variant had significantly higher cholesterol levels [35]. These variants also showed an association with higher BMI values in a study conducted among residents of Moscow, Russia [34].

The common intron variant rs1501299 has been associated with Type 2 diabetes in Saudi Arabian and Japanese populations [37]. In the Finnish population, this variant correlated with obesity [44], in the Indian population with low levels of adiponectin [45]. The GG genotype (common G allele) has been associated with favorable changes in adiponectin levels, insulin resistance, and lipid profile after two different dietary interventions [47].

### 3.2. Variants in RETN

This gene encodes a protein called resistin. Variants in the *RETN* gene correlate with signs of metabolic syndrome, T2DM, and BMI (Table 3).

The intron variant rs34788323 was found in our study in 21 heterozygous carriers and in one homozygous carrier (age 42, BMI 35.71, high levels of triglycerides, LDL, and glucose). This variant has previously been reported to correlate with higher levels of resistin in Iraqi women [49].

Our study identified two heterozygous C>A carriers of the intron variant rs377473014 and three C>T carriers. It has not previously been reported that both alleles of these variants are associated with metabolic disorders.

The 3′UTR rs3745368 was registered in 16 heterozygous carriers. Allele A of the rs3745368 variant was associated with lower levels of resistin in the Japanese population. It is assumed that the mechanism consists of an effect on the polyadenylation of RETN mRNA [51]. The ClinVar database reported that this variant is a risk factor for the development of DM2, insulin resistance, and arterial hypertension [52].

### 3.3. Variants in LEP

The leptin gene encodes a protein that is expressed by white adipocytes and secreted into the blood (Table 4).

Among young adults with abdominal obesity, one homozygous carrier and four heterozygous carriers of the intron variant rs17151914 of the LEP gene were identified. A synonymous variant rs138908051 was identified in one heterozygous carrier (one participant, age 27, BMI: 24.67, high LDL and high cholesterol). The ClinVar database provides conflicting results on the clinical significance of this variant [53]. Five patients were identified as heterozygous carriers of the 3′UTR rs62481073 variant, all of which had abnormal levels of LDL, cholesterol, and glucose.

### 3.4. Variants in APLN

The *APLN* gene codes for a preproprotein that is subsequently cleaved and activated in the endoplasmic reticulum (Table 5). Apelin plays an important part in the regulation of many biological functions, including insulin secretion [54].

The 3′UTR rs3115758 was registered in the homozygous form (TT) in one carrier (BMI 27.4, high LDL and cholesterol).

The intron variant rs909656 was identified in four heterozygous carriers in our study and in one homozygous carrier. In the homozygous variant (AA), the subject had a BMI of 32.64 and high levels of glucose and LDL.

Only one patient was a carrier of allele A of the intron variant rs375839749 (BMI 33, very high cholesterol, LDL and TG, and high glucose). None of the *APLN* gene variants that we identified have been previously described as correlated with metabolic disorders.

### 3.5. Variants in APLNR

Studies on *APLNR* gene variants (Table 6) have not detected any associations with diabetes mellitus or obesity [55]. Some variants in the *APLNR* gene correlate with the risk of arterial hypertension [56].

A rare rs199589565 variant was identified in one patient in our study (BMI 28, with normal biomarkers). A rare synonymous variant rs753649420 was also found in one patient (BMI 28, high levels of LDL and glucose). A rare variant 5’UTR rs368731106 was registered in three patients (BMI > 31). No association of these variants with obesity has previously been reported.

### 3.6. Variants in ADRB3

*ADRB3* is expressed mostly in adipose tissue [57]. Several variants of the *ADRB3* gene are associated with the development of obesity and T2DM (Table 7).

The rare missense variants rs746415961 and rs200163984 were identified once in a heterozygous variant. A synonymous variant rs549473233 was identified in one heterozygous carrier with a BMI of 40.15 and high glucose and LDL levels.

The variants rs4997 and rs4994 are in complete linkage disequilibrium (r^2^ = 1). Three homozygous carriers of these variants have been identified. The misssense variant of rs4994 has been well studied and was first described as being associated with obesity in the Japanese population [60]. More recent studies have shown an association of this variant with obesity and T2DM in populations of East Asia [59], Iran [58], and the indigenous population of the Amazon [61]. The mechanism by which this variant acts is thought to be an alteration in receptor function leading to the decreased expression of hormone-sensitive lipase (HSL), most likely causing obesity [62].

### 3.7. Variants in GCG

The missense variant rs150179526 was identified in five patients with hyperlipidemia and hyperglycemia (Table 8).

The intron variant rs5649 was identified in five heterozygous carriers with normal biomarker levels, while the only homozygous carrier of the T allele of the rs5646 variant was a man with a BMI of 33 and with a very high level of TG and total cholesterol, LDL, and glucose.

Allele A of the rs5645 was found by us in 11 heterozygous carriers. This variant was previously described as being associated with clopidogrel treatment resistance in the Han Chinese population [63].

### 3.8. Variants in GIP

Only one rare variant, rs117649535, was identified in the *GIP* gene (Table 9) in one heterozygous carrier with a BMI of 34.5 and high blood glucose.

The common variant rs2291725 in the GIP gene was previously described as being associated with a high risk of CVD [64].

### 3.9. Variants in PPY and PYY

The rs771706654 variant that gives rise to a stop codon was identified in the PPY gene in the one heterozygous carrier with a BMI of 27 and a high LDL level (Table 10).

We did not identify any rare variant in the *PYY* gene in young adults with abdominal obesity in our study (Table 11).

### 3.10. Variants in SCT

Secretin expression activates brown adipose tissue and reduces central responses to appetizing food [65]. The missense variant rs376423879 in the SCT gene was identified in one heterozygous carrier with a BMI of 27 and high LDL and total cholesterol (Table 12).

The intron variant rs187861364 was found in one heterozygous carrier (BMI 33.6, with a normal level of biomarkers). Another intron variant rs780568458 was found in one heterozygous and three homozygous carriers, all of whom had high glucose levels.

### 3.11. Variants in NAMPT

This gene encodes an enzyme that catalyzes the condensation of nicotinamide with 5-phosphoribosyl-1-pyrophosphate. The enzyme acts as a cytokine and adipokine, and its secreted form is known as visfatin [66].

The intron variant rs70937087 was identified in one heterozygous carrier with a BMI of 29.9 and a normal level of biomarkers (Table 13). Another intron variant rs778300482 was identified in a heterozygous carrier with elevated levels of cholesterol, LDL, and blood glucose.

Two other intron variants, rs144888107 and rs41430346, were identified in four and six heterozygous carriers, respectively. None of the rare NAMPT variants discovered have previously been described as being associated with obesity.

### 3.12. Variants in GHRL

The *GHRL* gene encodes a preproprotein that is later cleaved, thereby yielding ghrelin and obestatin [67]. One of the ghrelin effects related to weight changes is an alteration of eating behavior; for example, the variant rs696217 has been associated in bulimia nervosa (Table 14).

The 3′UTR variant rs369305953 was identified in one carrier with a BMI of 30 and with normal biomarkers in the homozygous variant. The intron variant rs4684677 in the *GHRL* gene was identified in four patients in the heterozygous variant. One of the patients carrying this variant had a BMI of 49.

Rare missense variants rs139997338 and rs760055038 were found in the *GHRL* gene in one case in patients with abdominal obesity.

It was previously described that the missense variant rs4684677 is associated with obesity in the European population [68]. In our study, this variant was found in 24 carriers in the heterozygous variant and in 1 carrier in the homozygous variant (female, age 38, BMI 30.8, with normal biomarkers).

The missense variant rs696217 was identified in 34 carriers in the heterozygous variant and in 2 in the homozygous variant. It has previously been described that this variant is associated with obesity in the Japanese population [70], with the effect of sleep duration on obesity in adolescence [71], with resistance to weight loss in Finnish diabetic patients [72], and with bulimia nervosa [69].

One variant leading to a premature stop codon, rs139684563, was identified in two carriers in a heterozygous variant.

### 3.13. Variants in INS

The *INS* gene codes for the hormone insulin, which is responsible for the modulation of carbohydrate and lipid metabolism [73]. Some mutations in the *INS* can lead to specific subtypes of diabetes, such as maturity onset diabetes of the young (MODY) or neonatal diabetes [74]. Rare and common variants that were identified in our study are presented in Table 15.

In the *INS* gene, two rare intron variants were identified. Rs41275198 was found in one carrier in the heterozygous variant (female, age 43, BMI 26 and with a high level of LDL), and rs201659391 was found in four carriers in the heterozygous variant.

The only heterozygous carrier of the synonymous variant rs11564720 was a male (age 43, BMI 27) with high levels of TG, cholesterol, and LDL.

The 5’UTR variant of the *INS* gene was found in one heterozygous carrier (female, age 40, BMI 31, with high levels of cholesterol, LDL and glucose).

### 3.14. Variants in FTO

The *FTO* gene codes for a protein that plays an important part in the development of obesity and T2DM [79]. Variants that were identified in the *FTO* gene in our study are presented in Table 16.

Rare intron variants rs184850472, rs117546833, and rs144100465 were found in different carriers in heterozygous variants. All of these carriers had an abnormal lipid profile and high blood glucose levels.

In the rs144587536 and rs370874825 variants examined with rare alleles, normal levels of biomarkers were determined, despite abdominal obesity. A rare intron variant rs2287142 was identified in 16 heterozygous carriers.

A rare missense variant rs145884431 was identified in two heterozygous carriers with abdominal obesity and hyperglycemia. The missense variant rs150450891 was identified in four patients with high blood glucose and two patients with an abnormal lipid profile.

The 3′UTR variant rs375031347 and the 5’UTR variant rs567718105 were found in two different carriers in a heterozygous form. Neither of these two variants has previously been described as correlated with metabolic disorders.

### 3.15. Variants in GLP1R

The *GLP1R* gene encodes a transmembrane receptor for glucagon-like peptide 1 [80,81]. Rare and common variants that were identified in the *GLP1R* gene in our study are presented in Table 17.

In the study group, several rare variants were identified in the introns of the *GLP1R* gene. Allele A rs201068918 was detected in six carriers with abdominal obesity and heterozygous dyslipidemia.

The rs761387 variant was identified in 45 carriers in the heterozygous form and in two patients in the homozygous form. This variant has been reported to be associated with higher levels of GLP-1 and blood glucose, and also correlates with higher insulin levels after glipizide administration [88]. The T allele of the intron variant rs761386 was found in 22 examined individuals with abdominal obesity. Previously, in a study of the Chinese population, it was reported that this allele is associated with obesity [89].

In our study, the only missense variant in the *GLP1R* gene was rs3765467, which was detected in six patients in the heterozygous form. All of them had high LDL levels and high glucose levels. This variant has been associated with metabolic syndrome and a higher risk of DM2 in the Chinese population [85] and less effective treatment of DM2 with GLP1R agonists [90]. It has been suggested that this SNP disrupts GLP1R function by modifying the receptor and influencing its interaction with GLP1, which leads to a decrease in insulin secretion by B cells [85].

## 4. Discussion

Genetic predisposition [91,92] and the influence of environmental factors that contribute to the development of obesity form a complex of reasons for gaining excess weight [19]. Approximately 40–70% of the variation in excess body weight is explained by genetic factors [93]. In our study, all of the 203 young adults with abdominal obesity had some rare variant in the genes associated with obesity. The widest range of rare and common variants was presented in *ADIPOQ*, *FTO*, *GLP1R*, and *GHRL* genes. In previous studies, these genes were strongly associated with various phenotypes of obesity and metabolic disorders.

The *ADIPOQ* gene is expressed in adipose tissue exclusively. Pathogenic variants in this gene are associated with adiponectin deficiency [94]. According to studies, rare variants in *ADIPOQ* are associated with the level of adiponectin [94,95]. The rs17366653 variant is most significantly associated with the level of adiponectin SNP [31]. It has been shown that the minor allele reduces the level of adiponectin by 0.24 mg/mL. The contribution of low-frequency and rare variants of the *ADIPOQ* gene are important for obesity [96].

The *FTO* gene shows a strong association with the BMI, obesity risk, and T2DM [97]. We found 11 rare variants in the *FTO* gene not previously described in the literature as associated with the development of obesity. The analysis of rare and low-frequency variants in the *FTO* gene will expand information about the role of these variants in metabolic diseases.

The *GLP1R* gene plays an important part in the signaling cascades resulting in insulin secretion [98]. In the *GLP1R* gene, we found variants associated with dyslipidemia [82], resistance to liraglutide [83] and exenatide [84], metabolic syndrome [85], insulin levels [86], BMI [87], glucose levels [85,88], and obesity [89], according to previously studies.

The *GHRL* gene encodes the ghrelin-obestatin preproprotein. Ghrelin regulates hunger and pancreatic glucose-stimulated insulin secretion. Obestatin regulates adipocyte function and glucose metabolism [99]. In our study, the rs696217 variant in the *GHRL* gene was determined in 34 carriers in the heterozygous variant and in 2 in the homozygous variant. Previous studies have shown an association of this variant with the development of obesity and T2DM in different populations [70,72].

Based on the analysis of the significance of the variants, it is possible to construct a scale for the individual genetic risk of obesity. The risk of obesity can be modified, for example, through weight loss therapy [100]. Our daily exercise and nutritional choices have long-term consequences for our bodily function. Early identification of genetic risk can help improve quality of life and life expectancy [101]. In addition to hereditary factors, to calculate the risk of developing obesity, it is important to take into account information about the relationship between smoking, alcohol, education, exercise, sleep, smoking, and shift work and the development of obesity [102].

The limitation of study is the sample size: the sample sizes do not allow us to assess clinical-course features of the disease that are associated with various pathogenic variants in obesity-associated genes.

## 5. Conclusions

The use of targeted sequencing and clinical criteria makes it possible to identify carriers of rare clinically significant variants in a wide range of obesity-associated genes and to investigate their influence on phenotypic manifestations of abdominal obesity.

## Figures and Tables

**Table 1:** Main characteristics of the study group of young people with abdominal obesity (n = 203).

	Population Aged 25–44 Years	AO+ Aged 25–44 Years	*p*
Number of subjects, n	1512	203	-
Males/Females, %	44.4/55.6	43.3/56.7	0.660
Age, years	36.15 ± 6.038	38.67 ± 0.36	0.712
TC, mg/dL	194.3 ± 38.6	207.44 ± 2.92	0.001
HDL-C, mg/dL	51.5 ± 12.4	47.95 ± 0.94	0.001
LDL-C, mg/dL	121.8 ± 33.9	130.08 ± 2.44	0.001
TGs, mg/dL	104.3 ± 75.2	147.08 ± 8.33	0.01
Glucose, mMol/L	5.6 ± 0.8	5.91 ± 0.08	0.066
Body mass index, kg/m^2^	26.05 ± 5.5	30.47 ± 0.33	0.001
Leptin, ng/mL	6845.5 ± 7507.1	10172 ± 0.653	0.001
Adiponectin, μg/mL	83.6 ± 113.6	61.6 ± 5.76	0.051

Continuous variables are presented as the mean ± standard deviation. AO+, with abdominal obesity; HDL-C, high-density lipoprotein cholesterol; LDL-C, low-density lipoprotein cholesterol; TC, total cholesterol; TGs, triglycerides.

**Table 2:** The genetic variants in the *ADIPOQ* gene.

dbSNP ID	Nucleotide Changes (NM_004797.4)	Minor Allele Frequency (gnomAD v3.1.2)	Minor Allele Frequency (RUSeq)	Associated Phenotype *	Database (ClinVar [28], LOVD [29], VarSome [30])
rs17366653	NM_004797.4:c.-8-24T>C	0.0131	0.0134	*ADIPOQ* levels [31,32]	VarSome (benign)
rs199668131	NM_004797.4:c.-8-12T>G	0.00004708	0.003028	-	VarSome (Uncertain Significance)
rs2241766	NM_004797.4:c.45T>G	0.1130	0.08606	T2DM, BMI [33,34,35]	LOVD (likely pathogenic) VarSome (likely benign)
rs143606172	NM_004797.4:c.164G>A	0.00008643	-	-	VarSome (Uncertain Significance)
rs1501299	NM_004797.4:c.214+62G>C	0.2978	-	T2DM, *ADIPOQ* levels [36,37]	VarSome (likely benign)
rs62625753	NM_004797.4:c.268G>A	0.004572	0.003604	T2DM, *ADIPOQ* levels [38]	ClinVar (likely benign) VarSome (benign)
rs17366743	NM_004797.4:c.331T>C	0.02859	0.01540	T2DM [39,40]	VarSome (benign)
rs4068	NM_004797.4:c.*65C>T	0.007772	0.006316	-	VarSome (likely benign)

T2DM: Type 2 diabetes mellitus, * an association is reported in the literature.

**Table 3:** The genetic variants in the *RETN* gene identified in our study.

dbSNP ID	Nucleotide Changes (NM_020415.4)	Minor Allele Frequency (gnomADv3.1.2)	Minor Allele Frequency (RUSeq)	Associated Phenotype *	Database Record (ClinVar, LOVD, VarSome)
rs3219177	NM_020415.4:c.118+39C>T	0.2107	0.2011	Higher *RETN* levels [48]	VarSome (benign)
rs34788323	NM_020415.4:c.196+30C>T	0.07987	0.06572	Higher *RETN* levels [49]	VarSome (benign)
rs377473014	NM_020415.4:c.196+47C>A	0.001605	-	T2DM, BMI [33,34,35]	VarSome (likely benign)
rs377473014	NM_020415.4:c.196+47C>T	0.0006830	-	-	-
rs10402265	NM_020415.4:c.197-16G>C	0.8341	0.8316	Higher *RETN* and glucose levels [50]	VarSome (Uncertain Significance)
rs3745368	NM_020415.4:c.*62G>A	0.03681	0.03670	Lower *RETN* levels [51]	ClinVar (risk factor) VarSome (benign)

T2DM: Type 2 diabetes mellitus, * an association is reported in the literature.

**Table 4:** The genetic variants in the *LEP* gene identified in our study.

dbSNP ID	Nucleotide Changes (NM_000230.3)	Minor Allele Frequency (gnomADv3.1.2)	Minor Allele Frequency (RUSeq)	Associated Phenotype *	Database (Clin-Var, LOVD, VarSome)
rs17151914	NM_000230.3:c.145-50C>T	0.01003	0.02392	-	ClinVar (benign) VarSome (likely benign)
rs138908051	NM_000230.3:c.165G>A	0.0001781	0.000	-	ClinVar (Conflicting interpretations) VarSome (likely benign)
rs62481073	NM_000230.3:c.*33C>T	0.004656	0.004636	-	ClinVar (Uncertain significance) VarSome (likely benign)

* an association is reported in the literature.

**Table 5:** The genetic variants in the *APLN* gene identified in our study.

dbSNP ID	Nucleotide Changes (NM_017413.5)	Minor Allele Frequency (gnomADv3.1.2)	Minor Allele Frequency (RUSeq)	Associated Phenotype *	Database (ClinVar, LOVD, VarSome)
rs3115758	NM_017413.5:c.*36G>T	0.07277	0.06532	-	VarSome (benign)
rs909656	NM_017413.5:c.*5+36C>A	0. 001327	0.005335	-	VarSome (likely benign)
rs375839749	NM_017413.5:c.67+8C>T	0. 001129	0.0009200	-	VarSome (benign)

* an association is reported in the literature.

**Table 6:** The genetic variants in the *APLNR* gene identified in our study.

dbSNP ID	Nucleotide Changes (NM_005161.6)	Minor Allele Frequency (gnomADv3.1.2)	Minor Allele Frequency (RUSeq)	Associated Phenotype *	Database (ClinVar, LOVD, VarSome)
rs199589565	NM_005161.6:c.707G>A	0.0005580	0.000	-	VarSome (likely benign)
rs753649420	NM_005161.6:c.513G>A	0.00003533	0.0005587	-	VarSome (likely benign)
rs948847	NM_005161.6:c.135C>A	0.5531	0. 05842	-	VarSome (likely benign)
rs368731106	NM_005161.6:c.-44G>C	0.0007431	0.004958	-	VarSome (likely benign)

* an association is reported in the literature.

**Table 7:** The genetic variants in the *ADRB3* gene identified in our study.

dbSNP ID	Nucleotide Changes (NM_000025.3)	Minor Allele Frequency (gnomADv3.1.2)	Minor Allele Frequency (RUSeq)	Associated Phenotype *	Database (ClinVar, LOVD, VarSome)
rs4997	NM_000025.3:c.1205+14G>T	0.07794	0.08712	ClinVar/benign	VarSome (benign)
rs746415961	NM_000025.3:c.1196G>T	0.000	-	-	VarSome (likely benign)
rs549473233	NM_000025.3:c.783C>T	0.00003139	0.0006127	-	VarSome (likely benign)
rs200163984	NM_000025.3:c.578C>T	0.0001841	0.0005952	-	VarSome (likely benign)
rs4994	NM_000025.3:c.190T>C	0.07938	0.08967	T2DM, obesity [22,58,59]	ClinVar (benign) VarSome (benign)

T2DM: Type 2 diabetes mellitus, * an association is reported in the literature.

**Table 8:** The genetic variants in the *GCG* gene identified in our study.

dbSNP ID	Nucleotide Changes (NM_002054.5)	Minor Allele Frequency (gnomADv3.1.2)	Minor Allele Frequency (RUSeq)	Associated Phenotype *	Database (ClinVar, LOVD, VarSome)
rs150179526	NM_002054.5:c.472A>G	0.008273	0.004592	-	ClinVar (benign) VarSome (benign)
rs5649	NM_002054.5:c.254+5G>A	0.001003	0.01075	-	ClinVar (benign) VarSome (benign)
rs5646	NM_002054.5:c.92+12G>A	0.0007661	0.0005760	-	VarSome (likely benign)
rs5645	NM_002054.5:c.15C>T	0.02221	0.02813	resistance to clopidogrel [63]	VarSome (benign)

* an association is reported in the literature.

**Table 9:** The genetic variants in the *GIP* gene identified in our study.

dbSNP ID	Nucleotide Changes (NM_004123.3)	Minor Allele Frequency (gnomADv3.1.2)	Minor Allele Frequency (RUSeq)	Associated Phenotype *	Database (ClinVar, LOVD, VarSome)
rs55936433	NM_004123.3:c.*27G>T	0.2771	0.3020	-	VarSome (benign)
rs72833611	NM_004123.3:c.*26G>C	0.1320	0.1150	-	VarSome (benign)
rs6504587	NM_004123.3:c.351-42A>G	0.9999	1.000	-	VarSome (likely benign)
rs117649535	NM_004123.3:c.351-45C>T	0.007840	0.007528	-	VarSome (benign)
rs2291725	NM_004123.3:c.307A>G	0.5242	0.5188	higher risk of CAD [64]	VarSome (likely benign)
rs62078384	NM_004123.3:c.86+46G>A	0.5203	0.5129	-	VarSome (benign)

* an association is reported in the literature.

**Table 10:** The genetic variants in the *PPY* gene identified in our study.

dbSNP ID	Nucleotide Changes (NM_002722.5)	Minor Allele Frequency (gnomADv3.1.2)	Minor Allele Frequency (RUSeq)	Associated Phenotype *	Database (ClinVar, LOVD, VarSome)
rs231473	NM_002722.5:c.263+40A>G	0.5485	0.6235	-	VarSome (benign)
rs771706654	NM_002722.5:c.230C>T	0.0001059	0.0005747	-	VarSome (benign)

* an association is reported in the literature.

**Table 11:** The genetic variants in the *PYY* gene identified in our study.

dbSNP ID	Nucleotide Changes (NM_001394028.1)	Minor Allele Frequency (gnomADv3.1.2)	Minor Allele Frequency (RUSeq)	Associated Phenotype *	Database (ClinVar, LOVD, VarSome)
rs1058046	NM_001394028.1:c.215C>G	0.5485	0.6859	-	VarSome (benign)
rs229969	NM_001394028.1:c.109C>G	1.000	-	-	VarSome (likely benign)

* an association is reported in the literature.

**Table 12:** The genetic variants in the *SCT* gene identified in our study.

dbSNP ID	Nucleotide Changes NM_021920.4	Minor Allele Frequency (gnomADv3.1.2)	Minor Allele Frequency (RUSeq)	Associated Phenotype *	Database (ClinVar, LOVD, VarSome)
rs376423879	NM_021920.4:c.355C>T	0.0002084	-	-	VarSome (likely benign)
rs187861364	NM_021920.4:c.267-5T>C	0.004106	-	-	VarSome (benign)
rs780568458	NM_021920.4:c.71+31C>G	0.0002752	-	-	VarSome (benign)

* an association is reported in the literature.

**Table 13:** The genetic variants in the *NAMPT* gene identified in our study.

dbSNP ID	Nucleotide Changes (NM_005746.3)	Minor Allele Frequency (gnomADv3.1.2)	Minor Allele Frequency (RUSeq)	Associated Phenotype *	Database (ClinVar, LOVD, VarSome)
rs70937087	NM_005746.3:c.1366-8T>C	0.004980	0.009050	-	ClinVar (benign) VarSome (benign)
rs144888107	NM_005746.3:c.969+49C>G	0.01429	0.008333	-	VarSome (likely benign)
rs2302559	NM_005746.3:c.903A>G	0.6349	0.6588	-	VarSome (likely benign)
rs778300482	NM_005746.3:c.744-28A>G	0.000	-	-	VarSome (benign)
rs41430346	NM_005746.3:c.319-51G>C	0.01897	-	-	VarSome (benign)

* an association is reported in the literature.

**Table 14:** The genetic variants in the *GHRL* gene identified in our study.

dbSNP ID	Nucleotide Changes (NM_016362.5)	Minor Allele Frequency (gnomADv3.1.2)	Minor Allele Frequency (RUSeq)	Associated Phenotype *	Database (ClinVar, LOVD, VarSome)
rs369305953	NM_016362.5:c.*3G>A	0.0002712	0.0.0002966	-	VarSome (likely benign)
rs4684677	NM_016362.5:c.269A>T	0.06311	0.05895	Obesity [68]	CliVar (benign) VarSome (benign)
rs139997338	NM_016362.5:c.224G>A	0.00006977	0.0002962	-	VaSome (likely benign)
rs696217	NM_016362.5:c.214C>A	0.08006	0.07464	Obesity, Bulimia nervosa [69]	ClinVar (benign) VarSome (benign)
rs760055038	NM_016362.5:c.148C>T	0.0001085	0.0002969	-	VarSome (likely benign)
rs183593317	NM_016362.5:c.-29-7C>T	0.007265	0.007517	-	VarSome (benign)
rs139684563	NM_016362.5:c.-786G>A	0.006755	0.003019	-	VarSome (benign)

* an association is reported in the literature.

**Table 15:** The genetic variants in the *INS* gene identified in our study.

dbSNP ID	Nucleotide Changes (NM_000207.3)	Minor Allele Frequency (gnomADv3.1.2)	Minor Allele Frequency (RUSeq)	Associated Phenotype *	Database (ClinVar, LOVD, VarSome)
rs3842753	NM_000207.3:c.*22A>C	0.7202	0.7458	Insulin expression [75]	ClinVar (Benign) VarSome (likely benign)
rs3842752	NM_000207.3:c.*9C>T	0.2173	0.1997	Protective against T1D [76]	ClinVar (Benign-Likely benign) VarSome (benign)
rs41275198	NM_000207.3:c.188-10G>A	0.003191	0.000	-	ClinVar (Benign-Likely benign) VarSome (benign)
rs201659391	NM_000207.3:c.188-11C>T	0.001440	0.008113	-	VarSome (benign)
rs5506	NM_000207.3:c.187+11T>C	0.9993	1.000	-	ClinVar (Benign-Likely benign) VarSome (likely benign)
rs11564720	NM_000207.3:c.63A>G	0.0002312	0.0005931	-	ClinVar (Benign-Likely benign) VarSome (benign)
rs5505	NM_000207.3:c.-9C>T	0.01101	0.008662	-	ClinVar (Benign-Likely benign) VarSome (benign)
rs689	NM_000207.3:c.-17-6T>A	0.7214	0.7450	protective against T1DM/T2DM and IAA [76,77,78]	ClinVar (Benign) VarSome (likely benign)

T1DM: Type 1 diabetes mellitus, T2DM: Type 2 diabetes mellitus, * an association is reported in the literature.

**Table 16:** The genetic variants in the *FTO* gene identified in our study.

dbSNP ID	Nucleotide Changes (NM_001080432.3)	Minor Allele Frequency (gnomADv3.1.2)	Minor Allele Frequency (RUSeq)	Associated Phenotype *	Database (ClinVar, LOVD, VarSome)
rs375031347		0.0007271	0.002438	-	ClinVar (uncertain significance) VarSome (likely benign)
rs184850472	NM_001080432.3:c.45+29C>A	0.0004910	0.000	-	VarSome(likely benign)
rs116753298	NM_001080432.3:c.99C>T	0.0002557	-	-	ClinVar (Benign-Likely benign) VarSome (benign)
rs145884431	NM_001080432.3:c.487G>A	0.002617	0.002372		ClinVar (conflicting interpretation) VarSome (benign)
rs150450891	NM_001080432.3:c.601G>A	0.001038	0.007701	-	Clinvar (uncertain significance) VarSome (likely benign)
rs62033438	NM_001080432.3:c.895+37A>G	0.3711	0.3715	-	VarSome (benign)
rs11076004	NM_001080432.3:c.1119+31G>A	0.4150	0.3984	-	VarSome (benign)
rs144587536	NM_001080432.3:c.1120-45A>G	0.0007203	0.0005949	-	VarSome (likely benign)
rs117546833	NM_001080432.3:c.1239+24G>A	0.0001861	0.0005935	-	VarSome (benign)
rs370874825	NM_001080432.3:c.1239+32T>G	0.000	0.0005938	-	VarSome (likely benign)
rs144100465	NM_001080432.3:c.1239+22454G>A	0.004061	0.003961	-	VarSome (likely benign)
rs2287142	NM_001080432.3:c.1239+22488G>A	0.02829	0.02925	-	VarSome (benign)
rs567718105	NM_001080432.3:c.125A>G	0.0006077	0.003189	-	ClinVar (uncertain significance) VarSome (likely benign)

* an association is reported in the literature.

**Table 17:** The genetic variants in the *GLP1R* gene identified in our study.

dbSNP ID	Nucleotide Changes (NM_002062.5)	Minor Allele Frequency (gnomADv3.1.2)	Minor Allele Frequency (RUSeq)	Associated Phenotype *	Database (ClinVar, LOVD, VarSome)
rs10305420	NM_002062.5:c.20C>T	0.3921	0.3250	Dyslipidemia [82], resistance to liraglutide [83] and exenatide [84]	VarSome (benign)
rs201068918	NM_002062.5:c.283+34G>A	0.007481	0.007514	-	VarSome (benign)
rs3765468	NM_002062.5:c.390G>A	0.1047	0.1104	-	VarSome (benign)
rs3765467	NM_002062.5:c.392G>A	0.002684	0.01510	Metabolic syndrome/T2DM [85], insulin levels [86]	VarSome (benign)
rs6918287	NM_002062.5:c.399A>G	0.9887	0.9760		VarSome (Likely benign)
rs61754624	NM_002062.5:c.501C>T	0.0006594	0.005028	-	ClinVar (Likely benign) VarSome (benign)
rs6923761	NM_002062.5:c.502G>A	0.3301	0.2931	BMI and metabolic parameters [87]	LOVD (Uncertain significant) VarSome (benign)
rs10305457	NM_002062.5:c.509+16C>T	0.09757	0.1026	-	VarSome (Likely benign)
rs2235868	NM_002062.5:c.526A>C	0.5176	-	-	VarSome (benign)
rs200132876	NM_002062.5:c.774G>A	0.000007744	0.0005537	-	VarSome (Likely benign)
rs1042044	NM_002062.5:c.780A>C	0.5577	0.5742		VarSome (benign)
rs761387	NM_002062.5:c.884+43A>G	0.1013	0.1421	GLP-1 and glucose levels [88],	VarSome(benign)
rs10305492	NM_002062.5:c.946G>A	0.01591	0.01657	Insulin secretion-, T2DM, glucose levels [85]	VarSome (benign)
rs761386	NM_002062.5:c.955-17C>T	0.03038	0.05365	Obesity [89]	VarSome (Uncertain significant)
rs10305494	NM_002062.5:c.1044-37G>T	0.001031	0.007190	-	VarSome (benign)
rs12212036	NM_002062.5:c.1122C>T	0.005851	0.004860	-	ClinVar (Benign) VarSome (benign)
rs1126476	NM_002062.5:c.1200A>C	0.4744	0.5135	-	VarSome (benign)

T2DM: Type 2 diabetes mellitus, * an association is reported in the literature.

## Data Availability

The data presented in this study are available on request from the corresponding author.
